# Quality and Usability of Prostate Cancer Information Generated by Artificial Intelligence Chatbots: A Comparative Analysis

**DOI:** 10.3390/cancers18060906

**Published:** 2026-03-11

**Authors:** Abdullah Al-Khanaty, Jordan Santucci, David Hennes, Niranjan Sathianathen, Carlos Delgado, Karan Sharma, Eoin Dinneen, Kieran Sandhu, David Chen, Renu Eapen, Daniel Moon, Gregory Jack, Jeremy Goad, Shankar Siva, Muhammad Ali, Damien Bolton, Nathan Lawrentschuk, Declan G. Murphy, Marlon Perera

**Affiliations:** 1 Division of Cancer Surgery, Peter MacCallum Cancer Centre, Melbourne 3052, Australia; abdullah.alkhanaty@petermac.org (A.A.-K.);; 2 Department of Surgery, Austin Health, University of Melbourne, Melbourne 3052, Australia; 3 Prostate Cancer Theranostics and Imaging Centre of Excellence (ProsTIC), Peter MacCallum Cancer Centre, Melbourne 3052, Australia; 4 Department of Urology, Austin Health & Olivia Newton John Cancer Centre, Heidelberg 3084, Australia; 5 Department of Urology, Royal Melbourne Hospital, The University of Melbourne, Melbourne 3052, Australia; 6 Department of Urology, St Vincent’s Hospital Melbourne, Melbourne 3052, Australia; 7 Sir Peter MacCallum Department of Oncology, The University of Melbourne, Melbourne 3052, Australia; 8 Department of Radiation Oncology, Peter MacCallum Cancer Centre, Melbourne 3052, Australia; 9 EJ Whitten Prostate Cancer Research Centre at Epworth Healthcare, Melbourne 3052, Australia

**Keywords:** artificial intelligence, prostate cancer, patient education, health information quality, chatbots

## Abstract

Background: Artificial intelligence chatbots are increasingly used by patients to obtain health information, including for prostate cancer. While these platforms offer accessible and conversational responses, concerns remain regarding the quality, usability, and clinical relevance of AI-generated content. This study comparatively evaluated patient-directed prostate cancer information generated by commonly used AI chatbots. Methods: Standardised prostate cancer-related prompts were developed using Google Trends and authoritative healthcare resources. Identical queries were submitted to five publicly accessible AI chatbots: ChatGPT 5.2, Google Gemini, Claude AI, Microsoft Copilot, and Perplexity. Responses were independently assessed by two blinded reviewers using the DISCERN instrument for information quality and the Patient Education Materials Assessment Tool for printable materials (PEMAT-P) for understandability and actionability. Inter-rater reliability was assessed using intraclass correlation coefficients (ICCs). Readability was evaluated using the Flesch–Kincaid Reading Ease score. Descriptive statistics were used for comparative and pooled analyses. Results: Overall information quality was moderate, with a pooled median (interquartile range [IQR]) DISCERN score of 56.5 (53.0–61.0). Higher mean DISCERN scores were observed for ChatGPT 5.2 and Microsoft Copilot, whereas lower scores were observed for Claude and Perplexity. PEMAT-P understandability was consistently high across platforms, with a pooled median (IQR) score of 91.7% (83.3–91.7%). In contrast, PEMAT-P actionability was uniformly poor, with a pooled median (IQR) score of 0% (0–0%). Readability analysis demonstrated moderate complexity, with a pooled median (IQR) Flesch–Kincaid Reading Ease score of 50.4 (49.2–52.5) and a median word count of 666 (657–1022). Inter-rater reliability was good for PEMAT understandability (ICC 0.841) and moderate for DISCERN (ICC 0.712). Conclusions: AI chatbots provide highly understandable but only moderately high-quality patient-directed prostate cancer information, with a consistent lack of actionable guidance. Although variation in content quality was observed across platforms, significant limitations remain in evidence transparency and practical patient support. Future development should prioritise integration of evidence-based resources and actionable decision-support tools to enhance the role of AI chatbots in prostate cancer education.

## Simple Summary

Artificial intelligence chatbots such as ChatGPT are increasingly used by patients to seek information about prostate cancer. In this study, we evaluated several commonly available AI chatbots to assess the quality, clarity, and usefulness of the information they provide. We found that while most chatbots explained prostate cancer in a clear and easy-to-understand manner, the overall quality of information was moderate. Importantly, very little practical guidance was provided to help patients understand what steps to take after receiving information. Differences in information quality were observed across platforms, but no chatbot consistently demonstrated high-quality, actionable patient guidance. These findings suggest that AI chatbots may be useful for basic education about prostate cancer but should not replace discussions with healthcare professionals or trusted patient information resources.

## 1. Introduction

Prostate cancer is the most diagnosed malignancy among men in many developed countries and remains a leading cause of cancer-related morbidity and mortality worldwide [1,2,3]. Its clinical course is heterogeneous, ranging from indolent disease suitable for active surveillance to aggressive cancer requiring multimodal therapy [4,5]. As a result, patients are frequently required to navigate complex information regarding diagnosis, treatment options, potential side effects, and long-term outcomes. High-quality, accessible patient-directed information is therefore essential to support informed decision-making and patient engagement. However, patients increasingly seek supplementary information outside traditional clinical encounters, particularly through online resources, which vary widely in quality, accuracy, and usability [6,7].

The recent proliferation of freely accessible artificial intelligence (AI)-driven chatbots and search platforms has positioned these tools as increasingly popular sources of health information. Large language models, including platforms such as ChatGPT, Google Gemini, Claude AI, Microsoft Copilot, and Perplexity, can generate natural language responses to health-related queries in a conversational format. These technologies offer the potential to address information gaps by delivering tailored explanations of disease processes, diagnostic pathways, lifestyle considerations, and treatment options. Early evidence suggests that AI chatbots may provide understandable and clinically appropriate information for prostate cancer patients, with several studies demonstrating broadly accurate responses that may support patient education following diagnosis [8,9,10].

More recent evaluations have examined AI-generated cancer screening information against evidence-based standards. Dedicated prostate cancer chatbot assessments have reported generally high-quality screening information, although gaps in completeness and clarity remain [11]. Recent studies have also demonstrated variable quality and readability in AI-generated prostate cancer information [12]. Comparative analyses of ChatGPT responses against guideline-based cancer screening recommendations have demonstrated substantial concordance but identified omissions and occasional hallucinated content [13]. Readability studies in urological settings further suggest that although chatbot outputs may exceed recommended literacy thresholds, they are often perceived as understandable by patients [14]. Collectively, these findings indicate considerable promise but also highlight variability in accuracy, completeness, readability, and actionability.

Despite this promise, important concerns remain regarding the reliability, transparency, and clinical utility of AI-generated health information. Large language models do not rely on curated medical knowledge bases in real time and may lack clear referencing, explicit discussion of uncertainty, or consistent alignment with contemporary clinical guidelines [15,16]. Evaluations across urological conditions have demonstrated mixed results, with generally good understandability but variable content quality and consistently poor actionability [17,18,19]. Furthermore, much of the existing literature has focused on single platforms, most commonly ChatGPT, without direct comparative assessment of the multiple AI chatbots currently accessible to the public [8,20].

Given the prevalence of prostate cancer and the complexity of its management, there is a critical need to assess whether commonly used AI chatbots can serve as reliable adjuncts to traditional patient education resources. Comparative evaluation across platforms is particularly important, as patients may engage with different AI tools that vary in underlying architecture, training data, and interface design.

The primary objective of this study was to systematically evaluate the quality, understandability, actionability, and readability of patient-directed prostate cancer information generated by widely accessible AI chatbots using validated assessment instruments, including DISCERN, the Patient Education Materials Assessment Tool for printable materials (PEMAT-P), and the Flesch–Kincaid readability formula. A secondary objective was to compare chatbot responses generated using publicly driven queries derived from Google Trends with clinically curated prompts informed by authoritative prostate cancer resources. This study aims to inform clinicians, patients, and developers regarding the current role of AI chatbots in prostate cancer education and to identify areas for future optimisation.

## 2. Methods

### 2.1. Study Design and Chatbot Selection

This cross-sectional comparative evaluation study was conducted between 15 January 2025 and 20 January 2025, with the primary objective of assessing the quality, understandability, actionability, and readability of prostate cancer information generated by widely accessible artificial intelligence chatbots in response to patient-oriented queries.

A descriptive analytical framework was employed. The evaluated platforms represented commonly used, publicly accessible large language model systems. Specifically, the chatbots assessed were: ChatGPT (version 5.2; OpenAI, https://chat.openai.com accessed on 15 January 2026), Google Gemini (Google DeepMind, https://gemini.google.com accessed on 15 January 2026), Claude (Anthropic, https://claude.ai accessed on 15 January 2026), Microsoft Copilot (Microsoft, https://copilot.microsoft.com accessed on 15 January 2026), and Perplexity (Perplexity AI, https://www.perplexity.ai accessed on 15 January 2026). All platforms were accessed using their freely available public interfaces during the study period. ChatGPT responses were generated using the publicly accessible ChatGPT web interface without Application Programming Interface access or internal research preview tools, reflecting the model behaviour available to users at the time of data collection.

### 2.2. Query Selection and Prompt Development

Query selection was conducted to reflect both real-world patient information-seeking behaviour and clinically relevant educational priorities in prostate cancer.

To identify commonly searched public queries, Google Trends data were analysed for the term “prostate cancer” over a 12-month period from 1 January 2025 to 31 December 2025, with search parameters set to Worldwide region, Web Search category, and All categories. To ensure broader contextual coverage, expanded query mapping was also performed for the related search terms “prostate specific antigen” and “prostate biopsy.” The complete list of search-associated terms identified during this mapping process is provided in Supplementary Table S1

From this broader pool of publicly searched terminology, candidate queries were reviewed and grouped into thematic clusters reflecting major patient information domains. Queries addressing similar informational themes were consolidated to avoid redundancy. Where multiple related search terms reflected the same underlying information need, representative patient-oriented questions were formulated to reflect the underlying search intent while maintaining clarity for chatbot prompting. The five most frequently associated and clinically relevant queries were then selected to represent commonly encountered public information-seeking priorities while ensuring coverage across distinct domains of prostate cancer understanding. These Google Trends-derived queries were:

1. “What is the prostate and what does it do?”
2. “What is prostate cancer?”
3. “What are the symptoms of prostate cancer?”
4. “What is a PSA test and what does PSA mean?”
5. “What are the treatment options for prostate cancer?”

To complement publicly derived searches, five clinically grounded patient-oriented questions were developed using authoritative prostate cancer educational resources, including Prostate Cancer Foundation Australia (including the Living with Prostate Cancer booklet), Cancer Council Australia (online materials and the Understanding Prostate Cancer booklet), and European Association of Urology patient information materials. These resources were reviewed in full by clinician authors.

Prompt development was not undertaken using a formal structured framework. Instead, commonly addressed patient questions and major informational themes were identified through review of these educational resources. Questions were selected to reflect frequently discussed aspects of prostate cancer across the patient journey, including epidemiology, risk factors, screening, disease classification, and treatment-related outcomes. Overlapping or conceptually redundant questions were consolidated to ensure broad coverage without duplication.

The final five clinically curated questions were:

1. “How common is prostate cancer in Australia?”
2. “What are the risk factors for prostate cancer, including family history and genetics?”
3. “Should I be screened for prostate cancer, and when?”
4. “What are the stages and grades of prostate cancer?”
5. “What are the side effects of prostate cancer treatments?”

All ten prompts were utilised exactly as written and were submitted in identical English wording across all chatbot platforms. Prompts were delivered as single-turn queries without iterative refinement, follow-up clarification, or prompt engineering to maintain methodological consistency and allow direct comparison of chatbot responses. The complete list of prompts and corresponding chatbot outputs is provided in the Supplementary Material to facilitate transparency and reproducibility (Supplementary Tables S2–S6).

The prompt set was intentionally limited to ten questions to balance breadth of topic coverage with methodological consistency across chatbot platforms. This number was considered sufficient to capture commonly encountered patient information needs spanning foundational domains of prostate cancer education, while maintaining a standardized and manageable framework for comparative evaluation of chatbot responses.

### 2.3. Query Submission Procedure

All ten standardized queries were individually submitted to each chatbot between 15 January 2025 and 20 January 2025 by a single investigator to maintain consistency and reduce user-induced variability. All prompts were manually entered using identical phrasing within a controlled data-collection timeframe to ensure uniform submission conditions across platforms.

To mitigate potential bias arising from search history, personalized web content, or prior interactions, the investigator utilized Incognito browser mode during all chatbot sessions. The browser cache and cookies were systematically cleared prior to each platform interaction. Chat histories were reset before submission of each new query to prevent contextual carryover effects. These measures were implemented to ensure that generated responses reflected the intrinsic performance of each chatbot at the time of access.

Responses were recorded exactly as generated, without follow-up prompts, clarifications, or conversational refinements, thereby preserving the raw, unaltered output of each platform.

All chatbot outputs were subsequently exported verbatim into a standardized document format. Platform identifiers, branding elements, hyperlinks, and metadata were removed to ensure de-identification. Each response was assigned a randomized alphanumeric code by an investigator not involved in outcome assessment. The coding key was concealed until completion of scoring.

### 2.4. Assessment of Quality, Understandability, and Actionability

All de-identified chatbot outputs were independently evaluated by two urologists with subspecialty expertise in uro-oncology. Neither reviewer was involved in prompt development, chatbot interaction, response extraction, or coding procedures.

Reviewers were blinded to chatbot identity and to each other’s assessments. Information quality was assessed using the DISCERN instrument, a validated 16-item tool designed to evaluate consumer health information related to treatment choices. Each item is scored on a 5-point Likert scale, with total scores ranging from 16 to 80, where higher scores indicate higher information quality.

Understandability and actionability were assessed using the Patient Education Materials Assessment Tool for printable materials (PEMAT-P). Understandability reflects how easily individuals of varying backgrounds can comprehend the material, while actionability evaluates the clarity of steps users can take based on the information provided. Scores are reported as percentages from 0 percent to 100 percent.

Scores were recorded independently without adjudication.

### 2.5. Readability Assessment

Readability of each chatbot response was evaluated using the Flesch–Kincaid Reading Ease formula, which estimates text complexity based on sentence length and word syllable count. Higher scores indicate easier readability. Word counts were recorded for each response.

### 2.6. Statistical Analysis

Descriptive statistics were used to summarize chatbot performance across quality, understandability, actionability, and readability domains. Mean values with ranges were reported for individual chatbot comparisons. Pooled analyses across all platforms were summarized using median values with interquartile ranges.

Given the exploratory design of the study, the limited number of prompts evaluated, and the descriptive objective of comparing chatbot outputs across platforms, formal inferential statistical testing between chatbots was not performed. Results are therefore presented using descriptive statistics to illustrate observed score patterns rather than statistically significant differences between platforms.

Inter-rater reliability between the two reviewers was assessed using the intraclass correlation coefficient (ICC). ICC analysis was performed for PEMAT understandability scores and DISCERN total scores to quantify agreement between raters. Reliability was interpreted using commonly accepted thresholds, with values below 0.50 indicating poor reliability, 0.50 to 0.75 moderate reliability, 0.75 to 0.90 good reliability, and greater than 0.90 excellent reliability.

ICC could not be calculated for the PEMAT actionability domain due to a high proportion of items being rated as not applicable, which resulted in insufficient variability for reliable estimation of agreement.

## 3. Results

### 3.1. Quality of Patient-Directed Prostate Cancer Information from AI Chatbots According to DISCERN Assessment

Across all five AI chatbots evaluated, the overall quality of patient-facing prostate cancer information was moderate. When all chatbot assessments were grouped together, the median (interquartile range [IQR]) DISCERN score was 56.5 (53.0–61.0) (Table 1). There was variability in performance between platforms (Table 2). Higher mean DISCERN scores were observed for ChatGPT 5.2 (60.0 [59–61) and Microsoft Copilot (61.0 [59–63]), whereas lower scores were observed for Claude (52.5 [51–54]) and Perplexity (53.5 [53,54]). Gemini demonstrated intermediate performance with the widest inter-assessor spread in DISCERN scores (mean 56.5, range 52–61).

Across DISCERN domains, scores were consistently higher for clarity of aims, relevance to patients, and acknowledgment of multiple treatment options. Lower scores were observed for provision of additional sources of support, clarity of information sources used to generate responses, discussion of uncertainty, and description of outcomes in the absence of treatment.

### 3.2. Understandability of Patient-Directed Prostate Cancer Information from AI Chatbots

PEMAT-P Understandability scores were high across all chatbots, with a pooled median (IQR) Understandability score was 91.7% (83.3–91.7%) (Table 1). ChatGPT 5.2, Gemini, and Copilot demonstrated uniformly high understandability scores of 91.7% with no inter-assessor variability. Claude had lower understandability scores (mean 79.2%, range 75.0–83.3%), driven by less consistent information chunking and limited use of visual cues. Perplexity demonstrated moderate variability between assessors, with a mean understandability score of 87.5% (range 83.3–91.7%). Strengths across platforms included use of plain language, logical sequencing, and avoidance of unnecessary calculations. Visual aids were rarely employed, limiting further gains in understandability.

### 3.3. Actionability of Patient-Directed Prostate Cancer Information from AI Chatbots

In contrast to understandability, PEMAT-P actionability was consistently poor across all platforms. The pooled median (IQR) PEMAT-P actionability score of all chatbot responses grouped together was 0% (0–0%) (Table 1). Common deficits included lack of explicit patient-directed instructions, absence of checklists or decision aids, and failure to break actions into manageable steps. ChatGPT 5.2, Claude, and Gemini provided no actionable guidance according to PEMAT-P criteria. Microsoft Copilot and Perplexity demonstrated minimal actionability, each achieving a mean (range) score of 10% (0–20%), reflecting occasional identification of a single patient action without accompanying stepwise guidance or tangible tools. Examples of minimal actionable statements identified in Microsoft Copilot and Perplexity responses, compared with purely informational outputs from other platforms, are provided in Supplementary Table S7.

### 3.4. Between-Chatbot Comparison

When assessed comparatively (Table 2), variation in score patterns was observed across platforms. ChatGPT 5.2 and Microsoft Copilot demonstrated relatively higher DISCERN scores combined with high understandability scores, whereas Claude demonstrated lower scores across these domains. Perplexity and Gemini demonstrated intermediate score patterns with variability between assessments. No chatbot demonstrated consistently strong performance across all three domains, with actionability remaining the key deficiency across platforms.

### 3.5. Sensitivity Analysis for Quality Assessment–Impact of Global Score

A sensitivity analysis excluding DISCERN Item 16 (overall quality rating) produced no material change in relative chatbot ranking or overall inferences. Median DISCERN scores decreased uniformly across platforms, confirming that observed differences were driven primarily by item-level content quality rather than the global assessment item alone.

### 3.6. Readability of AI-Generated Patient-Directed Prostate Cancer Information

Readability analysis demonstrated moderate text complexity across all AI chatbot platforms. The pooled median (interquartile range [IQR]) Flesch–Kincaid Reading Ease score was 50.4 (49.2–52.5) (Table 1), corresponding to approximately high school-level readability.

Across individual platforms (Table 2), Perplexity responses had the highest Reading Ease score (59.3), whereas Microsoft Copilot responses had the lowest Reading Ease score (47.1). ChatGPT 5.2, Gemini, and Claude demonstrated intermediate readability scores of 50.4, 52.5, and 49.2, respectively.

Response length varied substantially between platforms. The pooled median (IQR) word count was 666 (657–1022) (Table 1). Claude responses were the longest (1241 words), followed by Perplexity (1022 words), whereas ChatGPT 5.2, Gemini, and Microsoft Copilot generated responses of similar length (657, 666, and 656 words, respectively).

### 3.7. Inter-Rater Reliability

Inter-rater reliability between the two independent urologist assessors was good for PEMAT-P understandability, with an intraclass correlation coefficient (ICC) of 0.841. Agreement for DISCERN scoring was moderate, with an ICC of 0.712.

ICC could not be calculated for PEMAT-P actionability due to a high proportion of items being rated as not applicable, resulting in insufficient variability to generate a reliable estimate of agreement.

## 4. Discussion

This study provides a contemporary comparative evaluation of patient-directed prostate cancer information generated by widely accessible AI chatbots, demonstrating consistently high understandability, moderate overall information quality, and uniformly poor actionability. Although some platforms, including ChatGPT 5.2 and Microsoft Copilot, demonstrated comparatively higher content quality and clarity, no chatbot demonstrated consistently strong performance across all evaluated domains. These findings highlight a persistent gap between AI chatbots’ ability to present medical information in an accessible manner and their capacity to provide practical guidance that supports patient decision-making.

Overall information quality assessed by the DISCERN instrument was moderate, with strengths observed in clarity of aims, relevance, and coverage of treatment options. However, important deficiencies were noted in transparency regarding information sources, discussion of uncertainty, and provision of supportive resources. Similar concerns have been raised across multiple studies evaluating AI-generated urological health information, which have reported that while responses may appear broadly accurate, they frequently lack clear sourcing and nuanced guideline-based discussion [7,15,16,20].

The uniformly high PEMAT-P understandability scores observed in this study reflect the growing capacity of large language models to translate complex medical concepts into clear and structured explanations. This aligns with prior evaluations across both malignant and benign urological conditions suggesting that AI-generated information is often coherent and relatively easy to follow [8,21,22,23,24,25,26]. Prostate cancer-specific studies have also suggested that chatbot responses may be accurate and well received by patients, particularly when prompts encourage simplified language [9,10]. However, these benefits have frequently been accompanied by concerns regarding transparency, completeness, and clinical depth.

In contrast, PEMAT-P actionability remained consistently poor across all platforms in the present study, with most chatbots failing to provide explicit patient-directed steps, structured guidance, or decision-support tools. This finding is consistent with patterns reported across the broader literature. Evaluations of chatbot responses related to urological malignancies have demonstrated moderate to high information quality but limited actionability [17,18]. with similar limitations reported across renal cell carcinoma, kidney stones, benign prostatic hyperplasia, and pelvic floor disorders [21,27,28,29]. Collectively, these findings suggest that while AI chatbots can effectively explain medical concepts, they remain limited in translating information into practical patient-oriented guidance.

Several factors may contribute to this observation. Prompting strategy may influence chatbot outputs. In the present study, prompts were deliberately standardised and delivered as single-turn patient-oriented queries without follow-up clarification in order to simulate a typical initial information-seeking interaction and to maintain comparability across platforms. As a result, chatbot responses tended to provide explanatory information rather than explicit patient-directed guidance. It is plausible that more directive prompts, such as requesting step-by-step guidance, decision aids, or asking “what should I do next?”, could yield higher PEMAT-P actionability scores. Similarly, multi-turn conversational engagement may allow chatbots to refine responses and generate more structured patient guidance.

A further consideration is the suitability of PEMAT-P for evaluating conversational AI outputs. PEMAT-P was originally developed to assess static printable patient education materials, and some of its actionability criteria, such as the inclusion of visual aids, tangible tools, or clearly structured stepwise instructions, may not align perfectly with single-turn chatbot responses. The low actionability scores observed in this study may therefore reflect both limitations of the assessment tool in this context and genuine limitations in chatbot design. Chatbots are typically optimized to provide explanatory summaries rather than explicit behavioural instructions or decision-support frameworks when responding to broad patient questions. Nevertheless, from a patient education perspective, the absence of clear next-step guidance remains clinically relevant. Future research should explore the impact of targeted prompt engineering, iterative dialogue, and potentially adapted evaluation frameworks to better assess actionable guidance in AI-generated health communication.

Readability analysis in this study demonstrated moderate text complexity across platforms, with median Flesch–Kincaid Reading Ease scores corresponding approximately to high school-level readability. This contrasts with many earlier studies, which frequently reported college-level or advanced reading complexity for AI-generated health information [8,19,28,30,31]. The comparatively lower complexity observed in the present study may reflect improvements in contemporary language models or differences in prompt design aimed at patient-level communication.

However, readability should still be interpreted cautiously. Although the observed reading levels approximate high school-level text complexity, this remains above the health literacy level often recommended for patient education materials, which are commonly advised to target a sixth- to eighth-grade reading level. As such, while the information generated by chatbots was generally understandable according to structured assessment tools, it may still exceed the optimal reading level for some patient populations, particularly individuals with lower health literacy or limited familiarity with medical terminology. Prior prostate cancer-focused research has highlighted poor readability as a potential barrier to effective patient education, particularly given the established association between low health literacy and poorer cancer outcomes [8,32]. Importantly, however, improved readability did not translate into enhanced actionability in the present study, reinforcing that linguistic simplicity alone is insufficient to support effective patient engagement and decision-making. Accordingly, AI-generated health information should be considered a supplementary educational resource rather than a substitute for clinician-guided discussions or established patient information materials.

Comparative studies across AI platforms have demonstrated substantial variability in accuracy, comprehensiveness, readability, and usability. Some models have shown greater alignment with guideline-based content, while others produced more readable but less complete responses [10,19,29]. Our head-to-head comparison similarly observed variability between chatbots. Higher DISCERN scores were observed for ChatGPT 5.2 and Microsoft Copilot, although no platform demonstrated consistently strong performance across all evaluated domains. The consistency of these shortcomings across studies suggests systemic constraints in current generative AI systems rather than deficiencies of individual platforms.

Importantly, while most existing research has focused on general-purpose chatbots, emerging work has explored specialised AI systems designed specifically for prostate cancer education. A prostate cancer-focused chatbot (PROSCA) demonstrated improvements in patient knowledge and satisfaction in both pilot and randomised controlled trials, supporting the potential value of curated, condition-specific AI tools integrated with validated clinical information [33,34]. These findings suggest that targeted AI systems may overcome some limitations observed with broadly trained chatbots.

A major strength of this study is its structured, comparative assessment of multiple widely used AI chatbots using established evaluation frameworks. The integration of both publicly driven search queries and clinically informed prompts allowed examination of a diverse set of informational needs relevant to prostate cancer patients. Blinded independent review enhanced methodological robustness and reduced assessment bias. Importantly, this work extends beyond prior research by directly comparing common AI platforms, providing novel insight into relative performance and identifying consistent gaps in patient-directed information delivery.

Several limitations should be acknowledged. The chatbot prompts were generated using a combination of high-frequency public search queries and content derived from established prostate cancer education resources. While this approach was designed to reflect both real-world information-seeking behaviour and clinically relevant topics, it may not encompass the full range of individualised concerns experienced by patients across different stages of prostate cancer. The relatively small number of prompts assessed may further limit the breadth of information evaluated and reduce the generalisability of the findings. Important patient-relevant domains, including advanced or metastatic disease, recurrence, survivorship, psychosocial impact, and decisional conflict, were not specifically addressed by the selected prompts. Additionally, patients and caregivers were not directly involved in the development of input queries, which may restrict the extent to which the assessment reflects patient-prioritised information needs.

Prompting strategy may also influence chatbot responses. In this study, prompts were deliberately standardised and delivered as single-turn queries without follow-up clarification in order to simulate a typical initial patient information-seeking interaction and to maintain comparability across platforms. However, alternative prompting approaches, including more directive prompts or multi-turn conversational engagement, may yield different outputs and potentially greater actionable guidance. As such, the selected prompt set may not fully capture the breadth of chatbot capabilities or the dynamic nature of real-world patient–chatbot interactions.

Although validated instruments were employed to evaluate content quality and usability, these tools primarily focus on structural and informational elements and may not fully capture aspects such as conversational tone, emotional sensitivity, or personal relevance, which are particularly important in cancer-related communication. In addition, the PEMAT-P was originally designed to evaluate static written patient education materials rather than conversational artificial intelligence outputs, which may influence how actionability is captured in chatbot-generated responses. While inter-rater reliability was formally assessed, some domains, particularly actionability, demonstrated floor effects that constrained variance-based agreement estimates.

Importantly, this study did not formally evaluate factual accuracy against guideline-concordant standards or systematically assess the presence of hallucinated, outdated, or dynamically changing information. Large language model outputs are probabilistic and may occasionally generate incomplete or incorrect information. These risks were not specifically evaluated in the present analysis but remain important considerations when interpreting AI-generated health content. Furthermore, the readability levels observed in this study corresponded approximately to high school-level text complexity, which may exceed the reading level recommended for some patient education materials. As such, although the chatbot outputs demonstrated high understandability according to structured assessment tools, the information may still be challenging for individuals with lower health literacy or limited familiarity with medical terminology. These considerations highlight the importance of viewing AI chatbot outputs as supplementary educational resources rather than replacements for clinician-guided discussions or established patient information materials.

Finally, the analysis was confined to freely available versions of selected AI chatbots evaluated at a single time point. Given the rapid evolution of large language models, performance may differ in newer iterations or paid platforms with expanded capabilities. Although prompts were standardised and chat histories cleared before each interaction, variability in responses may still occur due to ongoing model updates and inherent response generation dynamics. In routine use, conversational history is often retained, which may influence chatbot outputs and warrants further investigation.

Future research should incorporate patient-reported perspectives, evaluate a broader range of chatbot platforms, and examine interactive multi-turn conversations that more closely reflect real-world use. Assessments tailored to varying health literacy levels and cultural contexts would further strengthen understanding of the role of artificial intelligence chatbots in prostate cancer education.

## 5. Conclusions

Widely accessible AI chatbots generate highly understandable but only moderately high-quality patient-directed prostate cancer information, with a consistent lack of actionable guidance. Although certain platforms outperform others in content quality, no chatbot demonstrates comprehensive strength across quality, understandability, and actionability domains. Future development should prioritise improved evidence transparency and patient-centred actionable tools to enhance the role of AI chatbots in prostate cancer education and shared decision-making.

## Figures and Tables

**Table 1:** Combined assessment of all artificial intelligence chatbots for prostate cancer information: Values represent median (interquartile range) scores after grouping all chatbot outputs together and pooling all assessor scores for each domain.

Variable	All Chatbots Grouped Together
DISCERN score, median (IQR)	56.5 (53.0–61.0)
PEMAT-P understandability score (%), median (IQR)	91.7 (83.3–91.7)
PEMAT-P actionability score (%), median (IQR)	0 (0–0)
Flesch–Kincaid Reading Ease score, median (IQR)	50.4 (49.2–52.5)
Word count, median (IQR)	666 (657–1022)

**Table 2:** Individual assessment of patient-facing prostate cancer information generated by each artificial intelligence chatbot. Values represent the mean of two independent consultant urologist assessments, with ranges shown to demonstrate inter-assessor variability.

Variable	ChatGPT	Claude	Gemini	Copilot	Perplexity
DISCERN score, average (range)	60.0 (59–61)	52.5 (51–54)	56.5 (52–61)	61.0 (59–63)	53.5 (53–54)
PEMAT-P understandability score (%), average (range)	91.7 (91.7–91.7)	79.2 (75.0–83.3)	91.7 (91.7–91.7)	91.7 (91.7–91.7)	87.5 (83.3–91.7)
PEMAT-P actionability score (%), average (range)	0 (0–0)	0 (0–0)	0 (0–0)	10.0 (0–20)	10.0 (0–20)
Flesch–Kincaid Reading Ease score	50.4	49.2	52.5	47.1	59.3
Word count	657	1241	666	656	1023

## Data Availability

The original contributions presented in this study are included in the article/Supplementary Material. Further inquiries can be directed to the corresponding author.

## Supplementary Materials

The following supporting information can be downloaded at: https://www.mdpi.com/article/10.3390/cancers18060906/s1, Table S1. Expanded Query Mapping and Authoritative Source Review Used to Inform Prompt Selection. Table S2. ChatGPT Responses to Standardized Prostate Cancer Prompts (Verbatim). Table S3. Google Gemini Responses to Standardized Prostate Cancer Prompts (Verbatim). Table S4. Claude Responses to Standardized Prostate Cancer Prompts (Verbatim). Table S5. Perplexity Responses to Standardized Prostate Cancer Prompts (Verbatim). Table S6. Microsoft Copilot Responses to Standardized Prostate Cancer Prompts (Verbatim). Table S7. Examples of Minimal Actionable Content Observed in Chatbot Responses.
