# Supplementary material for: Quality and Usability of Prostate Cancer Information Generated by Artificial Intelligence Chatbots: A Comparative Analysis
Source: Cancers (Basel). 2026 Mar 11;18(6):906. doi: 10.3390/cancers18060906 (PMC13024046; doi:10.3390/cancers18060906)
Supplement: Supplementary file 1 [file cancers-18-00906-s001.zip › cancers-4163390-supplementary.pdf]

Supplementary Table S1. Expanded Query Mapping and Authoritative Source Review Used to Inform Prompt Selection

Domain	Source / Search Term	Identified Associated Queries / Educational Headings
Google Trends	Search term: "Prostate cancer"	Symptoms prostate cancer; What is prostate; What is prostate cancer; Prostate symptoms; Prostate cancer treatment; Cancer de prostate; Prostate cancer news; PSA; PSA prostate cancer; Prostate cancer test; Treatment of prostate cancer; Prostate cancer cause; Cancer de la prostate; Prostate cancer UK; Symptoms of prostate cancer; Metastatic prostate cancer; Metastatic cancer; Prostate cancer screening; Prostate cancer news today
Google Trends	Search term: "Prostate Specific Antigen"	Prostate specific antigen test; PSA; What is prostate specific antigen; Prostate cancer; Prostate specific antigen range; Prostate specific antigen level; Prostate specific antigen high; Prostate specific antigen normal range; Prostate specific antigen meaning; Prostate meaning; Serum prostate specific antigen; PSA test; Prostate specific antigen levels; Prostate specific antigen blood test; Elevated prostate specific antigen; What is PSA; Prostate specific antigen means; Prostate specific antigen function; Prostate specific membrane antigen
Google Trends	Search term: "Prostate biopsy"	Biopsy of prostate; Biopsy for prostate; After prostate biopsy; MRI prostate biopsy; Prostate MRI; Biopsy on prostate; What is biopsy; Biopsy for prostate cancer; Transperineal; Transperineal biopsy; What is a prostate biopsy; Prostate biopsy procedure; Fusion prostate biopsy; Fusion biopsy; Transrectal prostate biopsy; Prostate biopsy results; Biopsy test
Authoritative Patient Resource	Prostate Cancer Foundation Australia – Living with Prostate Cancer (2024)	What is the prostate?; What is prostate cancer?; Symptoms of prostate cancer; Outlook and prognosis; Tests to diagnose prostate cancer; Understanding prostate cancer test results; What happens after diagnosis?; How is prostate cancer treated?; Common side effects of prostate cancer treatment; How do you know treatment has worked?
Authoritative Patient Resource	European Association of Urology (EAU) Patient Information – Prostate Cancer	The prostate; Prostate cancer; Stages of prostate cancer; Risk factors; Symptoms

Domain	Source / Search Term	Identified Associated Queries / Educational Headings
Authoritative Patient Resource	Cancer Council Australia – Understanding Prostate Cancer Booklet and Online Materials	What is prostate cancer?; How common is it?; What are the risk factors?; Does prostate cancer run in families?; What screening test should I have?; What are the symptoms?; Which health professionals will I see?

From this expanded pool of publicly searched terms and authoritative educational themes, ten standardized prompts were selected to ensure balanced coverage of anatomy, epidemiology, risk factors, screening, staging, treatment, and treatment-related side effects while minimizing redundancy.

Supplementary Table S2. ChatGPT Responses to Standardized Prostate Cancer Prompts (Verbatim)

Prompt	ChatGPT Response
Q1: What is the prostate and what does it do?	The prostate is a small gland below the bladder and in front of the rectum. It sits around the urethra (the tube you pass urine through). Its main job is to make fluid that nourishes and protects sperm, forming part of semen.
Q2: What is prostate cancer?	Prostate cancer happens when abnormal cells in the prostate grow uncontrollably, forming a malignant tumour. Some prostate cancers grow very slowly and may never cause problems, while others are more aggressive and can spread - this is why grade and stage matter.
Q3: What are the symptoms of prostate cancer?	Early prostate cancer often causes no symptoms. If symptoms occur, they can include urinary changes (slow stream, needing to urinate more often - especially at night), blood in urine or semen, or discomfort. Advanced cancer can cause symptoms like bone/back pain, fatigue, or weight loss. Importantly, urinary symptoms are often due to non-cancer causes (like benign enlargement), so symptoms don't confirm cancer - but they should be checked.
Q4: What is a PSA test and what does PSA mean?	PSA stands for prostate-specific antigen - a protein made by prostate cells. A PSA test is a blood test that measures PSA levels. PSA can rise with prostate cancer, but it can also rise due to benign enlargement, inflammation/infection, recent ejaculation, or procedures, so it's prostate-specific, not cancer-specific. An abnormal PSA usually means "needs further assessment," not "definitely cancer."
Q5: What are the treatment options for prostate cancer?	Treatment depends on how risky the cancer is, whether it has spread, and your overall health. Options can include active surveillance (monitoring low-risk cancer), surgery, radiation therapy, hormone therapy (ADT), and for more advanced disease sometimes

Prompt	ChatGPT Response
	chemotherapy, newer hormone tablets, targeted therapies, immunotherapy in selected cases, and supportive care.
Q6: How common is prostate cancer in Australia?	Prostate cancer is the most commonly diagnosed cancer in Australian men. In 2025, it's estimated there will be about 28,868 new diagnoses, and the average male lifetime risk is about 1 in 5 (19%) by age 85.
Q7: What are the risk factors for prostate cancer, including family history and genetics?	The biggest risk factor is age (risk rises notably after 50). Family history increases risk - especially if a father or brother had prostate cancer, particularly at a younger age. A smaller proportion of cases relate to inherited genetic factors; certain inherited variants (e.g., BRCA1/BRCA2) can increase risk and may be linked to more aggressive disease in some men.
Q8: Should I be screened for prostate cancer, and when?	In Australia, PSA testing is generally recommended as a shared decision with your doctor - because there are benefits (finding significant cancer early) and downsides (false positives, anxiety, biopsies, and possible over-treatment of slow-growing cancers). If you choose testing: many Australian resources suggest PSA every 2 years from age 50-69 for average-risk men, and starting earlier (often age 40-45) for men with a strong family history or higher risk.
Q9: What are the stages and grades of prostate cancer?	Stage tells where the cancer is: confined to the prostate, spread locally, to lymph nodes, or to distant sites (metastatic). Doctors commonly use TNM staging (Tumour, Nodes, Metastasis). Grade tells how abnormal/aggressive the cells look under the microscope - reported as Gleason score and/or ISUP Grade Group 1-5 (higher grade group generally means faster-growing/more aggressive).
Q10: What are the side effects of prostate cancer treatments?	Side effects depend on the treatment and the person. Common ones include:\n- Surgery: urinary leakage and erectile dysfunction can occur (often improve over time, but not always).\n- Radiation therapy: urinary irritation (frequency/urgency), bowel changes (looser stools/rectal irritation), fatigue, and erection changes.\n- Hormone therapy (ADT): hot flushes, reduced libido/erections, fatigue, weight gain, mood changes, and bone thinning.

Supplementary Table S3. Google Gemini Responses to Standardized Prostate Cancer Prompts (Verbatim)

Prompt	Google Gemini Response
Q1: What is the prostate and what does it do?	The prostate is a small gland, roughly the size of a walnut, found only in men. It sits just below the bladder and surrounds the urethra (the tube that carries urine). Its main biological job is to produce seminal fluid, which nourishes and protects sperm as they travel during ejaculation.
Q2: What is prostate cancer?	Prostate cancer occurs when cells in the prostate gland develop mutations in their DNA. These changes cause the cells to grow and divide much faster than healthy cells. Instead of dying off as they should,

Prompt	Google Gemini Response
	these abnormal cells accumulate and form a malignant tumor. If left untreated, these cells can eventually invade nearby tissues or spread to other parts of the body.
Q3: How common is prostate cancer in Australia?	It is the most commonly diagnosed cancer in Australian men. In 2025/2026, it is estimated that approximately 28,800 men will be diagnosed annually. Statistically, about 1 in 5 men will be diagnosed with prostate cancer by the time they are 85. While it is common, it also has one of the highest survival rates if detected early.
Q4: What are the risk factors, including family history?	- Age: The risk increases significantly after age 50.\n- Family History: Your risk is doubled if a father or brother was diagnosed. It is five times higher if two or more close relatives are affected.\n- Genetics: Inherited gene mutations, such as BRCA1 or BRCA2 (often associated with breast cancer), or Lynch Syndrome, can increase the likelihood of developing more aggressive forms of the disease.
Q5: What is a PSA test and what does PSA mean?	PSA stands for Prostate-Specific Antigen, a protein produced by both normal and cancerous prostate cells. A PSA test is a blood test that measures the level of this protein. While a high level can indicate cancer, it can also be raised by an enlarged prostate (BPH) or an infection (prostatitis). It acts as a "smoke alarm" that tells doctors more investigation is needed.
Q6: Should I be screened, and when?	Australian guidelines recommend a risk-based approach rather than a "one-size-fits-all" rule:\n- Average risk: Men aged 50-69 should discuss two-yearly PSA testing with their GP.\n- High risk: Men with a family history or genetic risk should consider starting testing at age 40 or 45.\n- Aged 70+: Testing is usually based on individual health and life expectancy.
Q7: What are the symptoms?	Early-stage prostate cancer often has no symptoms at all. As the tumor grows, symptoms may include:\n- Difficulty starting urination or a weak/interrupted stream.\n- Needing to urinate more frequently, especially at night.\n- Blood in the urine or semen.\n- New pain in the back, hips, or pelvis (which can be a sign of advanced disease).
Q8: What are the stages and grades?	Doctors use two main ways to describe the cancer:\n- Staging (TNM): Describes how far the cancer has spread. Stages I and II are localized (inside the prostate); Stage III is locally advanced; Stage IV (metastatic) means it has spread to bones or other organs.\n- Grading (Gleason Score): Describes how aggressive the cells look. Scores range from 6 (slow-growing) to 10 (very aggressive). You may also hear ISUP Grade Groups 1-5, where 1 is the least aggressive.
Q9: What are the treatment options?	- Active Surveillance: Carefully monitoring slow-growing cancer with regular tests to avoid unnecessary side effects.\n- Surgery (Radical Prostatectomy): Removing the entire prostate gland.\n- Radiation Therapy: Using high-energy beams or radioactive "seeds" to kill cancer cells.\n- Hormone Therapy (ADT): Lowering testosterone to "starve" the cancer cells of the fuel they need to grow.

Prompt	Google Gemini Response
Q10: What are the side effects of treatment?	Because the prostate is near the nerves for sexual function and the muscles for bladder control, treatments can cause:\n- Urinary Incontinence: Leaking urine (often temporary).\n- Erectile Dysfunction: Difficulty getting or keeping an erection.\n- Hormonal Changes: Fatigue, hot flushes, and loss of libido (mostly with hormone therapy).\n- Bowel Changes: Urgency or irritation (mostly seen with radiation).

<u>Supplementary Table S4. Claude Responses to Standardized Prostate Cancer Prompts (Verbatim)</u>

Prompt	Claude Response
Q1: What is the prostate and what does it do?	The prostate is a walnut-sized gland that sits just below the bladder in men. It wraps around the urethra, which is the tube that carries urine out of the body. The prostate's main job is to produce fluid that mixes with sperm to create semen. This fluid nourishes and protects sperm, helping them survive and move during ejaculation. The prostate also contains muscles that help push semen through the urethra during orgasm.
Q2: What is prostate cancer?	Prostate cancer occurs when cells in the prostate gland start growing abnormally and out of control. In a healthy prostate, cells grow, divide, and die in an organized way. In prostate cancer, this process breaks down - cells multiply when they shouldn't and don't die when they should. These abnormal cells can form a tumor. Most prostate cancers grow slowly and may not spread beyond the prostate, but some types are more aggressive and can spread to other parts of the body, particularly bones and lymph nodes.
Q3: What are the symptoms of prostate cancer?	Early-stage prostate cancer typically has no symptoms at all, which is why screening is important. When symptoms do appear, they often occur because the cancer has grown large enough to affect the urethra or has spread beyond the prostate. Symptoms may include difficulty starting urination, weak urine flow, frequent urination (especially at night), blood in urine or semen, pain or burning during urination, discomfort in the pelvic area, and erectile dysfunction. However, many of these symptoms can also be caused by non-cancerous conditions like benign prostatic hyperplasia (BPH), so they don't necessarily mean you have cancer.
Q4: What is a PSA test and what does PSA mean?	PSA stands for Prostate-Specific Antigen, a protein produced by both normal and cancerous prostate cells. A PSA test measures the level of this protein in your blood. Higher PSA levels can indicate prostate cancer, but they can also be elevated due to other conditions like prostate infections, inflammation, or benign enlargement. That's why a high PSA doesn't automatically mean you have cancer - it's more

Prompt	Claude Response
	of a warning sign that warrants further investigation. Your doctor will consider your PSA level along with other factors like your age, prostate size, and how your PSA changes over time.
Q5: What are the treatment options for prostate cancer?	Treatment depends on factors like the cancer's stage and grade, your age, overall health, and personal preferences. Options include:\nActive surveillance: Closely monitoring slow-growing cancers without immediate treatment, using regular PSA tests and biopsies.\nSurgery (radical prostatectomy): Removing the entire prostate gland and some surrounding tissue.\nRadiation therapy: Using high-energy beams to kill cancer cells, either from outside the body (external beam) or through radioactive seeds placed in the prostate (brachytherapy).\nHormone therapy: Blocking testosterone, which fuels prostate cancer growth, often used for advanced cancer or combined with radiation.\nChemotherapy: Used primarily for advanced cancer that has spread or doesn't respond to hormone therapy.\nNewer treatments: Including immunotherapy, targeted therapy, and focal therapies that treat only part of the prostate.
Q6: How common is prostate cancer in Australia?	Prostate cancer is the most commonly diagnosed cancer in Australian men. Each year, approximately 24,000 Australian men are diagnosed with prostate cancer, making it roughly one in every five cancer diagnoses in men. It's also the second most common cause of cancer death in Australian men after lung cancer. About 1 in 6 Australian men will be diagnosed with prostate cancer by age 85. The good news is that survival rates are high - around 96% of men diagnosed with prostate cancer survive at least 5 years, and many live much longer, especially when the cancer is detected early.
Q7: What are the risk factors for prostate cancer, including family history and genetics?	Several factors increase your risk of developing prostate cancer:\nAge: Risk increases significantly after age 50, with most cases diagnosed in men over 65.\nFamily history: Having a father or brother with prostate cancer more than doubles your risk. The risk is even higher if multiple family members were affected or if they were diagnosed at a younger age.\nGenetics: Inherited gene mutations, including BRCA1 and BRCA2 (the same genes linked to breast cancer), can increase prostate cancer risk. Lynch syndrome is another genetic condition that raises risk.\nEthnicity: Men of African descent have higher rates and tend to develop more aggressive forms at younger ages.\nOther factors: Obesity and diet may play a role, though the evidence is less clear than for other risk factors.
Q8: Should I be screened for prostate cancer, and when?	Prostate cancer screening is a personal decision you should make with your doctor, as it involves weighing benefits against potential harms. In Australia, there's no national screening program because screening can detect slow-growing cancers that may never cause problems, potentially leading to unnecessary treatment and side effects.\nGeneral guidelines: Men at average risk should discuss screening starting around age 50. Men at higher risk (family history, African ancestry, or genetic mutations) should start the conversation

Prompt	Claude Response
	earlier, around age 40-45.\nWhat screening involves: Typically a PSA blood test and sometimes a digital rectal exam (DRE). Elevated results may lead to an MRI or biopsy.\nThe key is informed decision-making - understanding that screening can save lives by catching aggressive cancers early, but can also lead to overdiagnosis and overtreatment of cancers that would never have caused harm.
Q9: What are the stages and grades of prostate cancer?	Staging and grading help determine how serious the cancer is and guide treatment decisions.\nGrading (Gleason Score/Grade Group): This describes how abnormal the cancer cells look under a microscope. The Gleason score ranges from 6 to 10, but doctors increasingly use Grade Groups 1-5. Lower grades (Group 1, Gleason 6) look more like normal cells and grow slowly. Higher grades (Group 5, Gleason 9-10) look very abnormal and are more aggressive. This tells you how the cancer is likely to behave.\nStaging: This describes how far the cancer has spread. Stage I and II cancers are confined to the prostate. Stage III has grown through the prostate capsule into nearby tissue. Stage IV has spread to lymph nodes, bones, or distant organs. Staging is determined using imaging tests like MRI, CT, or bone scans, combined with biopsy results. Together, stage and grade help predict outcomes and determine the best treatment approach.
Q10: What are the side effects of prostate cancer treatments?	Different treatments carry different side effects, though modern techniques have reduced their severity:\nSurgery side effects: Urinary incontinence (leakage) is common initially but usually improves over time. Erectile dysfunction affects many men, though nerve-sparing techniques can help preserve function. Less common issues include urinary strictures and, rarely, bowel injury.\nRadiation therapy side effects: Bowel problems (diarrhea, urgency, bleeding) and urinary irritation during and after treatment. Erectile dysfunction can develop gradually over time. Fatigue is common during treatment.\nHormone therapy side effects: Hot flashes, loss of libido, erectile dysfunction, fatigue, mood changes, loss of muscle mass, weight gain, and long-term risks of osteoporosis and cardiovascular issues.\nActive surveillance: No immediate treatment side effects, but can cause anxiety about living with untreated cancer.\nMany side effects are temporary or can be managed with medications, physical therapy, or lifestyle changes. Your medical team can help you understand which side effects are most likely with your specific treatment plan and how to manage them.

<u>Supplementary Table S5. Perplexity Responses to Standardized Prostate Cancer Prompts (Verbatim)</u>

Prompt	Perplexity Response
Q1: What is the prostate and what does it do?	The prostate is a small gland that sits just below the bladder in people assigned male at birth. It wraps around the urethra, the tube that carries urine from the bladder out through the penis. Its main job is to make some of the fluid in semen. This fluid helps sperm move and survive, which is important for fertility.
Q2: What is prostate cancer?	Prostate cancer happens when cells in the prostate gland start to grow in an uncontrolled way and form a tumour. These cancer cells can sometimes spread to nearby tissues or to other parts of the body, such as lymph nodes or bones. Some prostate cancers grow very slowly and may never cause serious problems, while others are faster growing and need active treatment.
Q3: What are the symptoms of prostate cancer?	Early prostate cancer often has no symptoms at all. Many men are diagnosed because of a blood test (PSA) or an abnormal prostate exam. When symptoms do occur, they can include: Needing to urinate more often, especially at night; A weak or slow urine stream, or difficulty starting or stopping; Burning or pain when passing urine; Blood in the urine or semen; Pain in the lower back, hips, or bones (usually in more advanced disease). These symptoms can also be caused by non cancer conditions like benign enlarged prostate or infection, so they always need medical assessment.
Q4: What is a PSA test and what does PSA mean?	PSA stands for "prostate specific antigen." It is a protein made by both normal and cancerous prostate cells. A PSA test is a simple blood test that measures how much PSA is in the blood. Higher than expected PSA levels can be a sign of prostate cancer, but they can also be raised by other conditions such as benign prostate enlargement, infection, or recent ejaculation. A high PSA does not automatically mean cancer; it is a signal that further tests may be needed.
Q5: What are the treatment options for prostate cancer?	Treatment depends on how aggressive the cancer is, whether it has spread, and on the person's age, health, and preferences. Common options include: Active surveillance or watchful waiting (regular monitoring, no immediate treatment); Surgery to remove the prostate (radical prostatectomy); Radiation therapy (external beam or internal "seed" brachytherapy); Hormone therapy to lower testosterone and slow cancer growth; Chemotherapy and newer drugs such as targeted therapy or immunotherapy, mainly for advanced cancer. Often, treatments are combined. The care team will explain which options best match the stage and risk of the cancer.
Q6: How common is prostate cancer in Australia?	Prostate cancer is one of the most common cancers diagnosed in Australian men. It is a leading cause of cancer in this group, but many men with prostate cancer live for many years, especially when it is found early. Australia has relatively high detection rates because of widespread PSA testing and an ageing population.
Q7: What are the risk factors, including family history and genetics?	Things that increase the risk of prostate cancer include: Age: Risk rises after about 50, and most cases occur in older men. Family history: Having a father, brother, or son with prostate cancer roughly doubles the risk, especially if they were diagnosed young. Genetic changes:

Prompt	Perplexity Response
	Inherited gene faults, such as in BRCA1 or BRCA2 (also linked with breast and ovarian cancer), can raise the risk. Ethnicity: Men of African ancestry have a higher risk and often more aggressive disease; risk is lower in some Asian populations. Other factors: Obesity and lack of exercise may be linked to a higher risk of aggressive prostate cancer. Having one or more risk factors does not mean someone will definitely get prostate cancer, but it can influence when to start discussions about testing.
Q8: Should I be screened for prostate cancer, and when?	There is no single rule that fits everyone, so screening should be a shared decision with a doctor. Screening usually means doing a PSA blood test, with or without a digital rectal exam. General guidance often used in practice: Men at average risk: consider discussing PSA testing from around age 50. Men at higher risk (strong family history, known gene mutation, or certain ethnic backgrounds): consider talking to a doctor earlier, for example around age 40–45. Screening can find cancers early, but it can also detect slow growing cancers that may never cause harm, leading to worry and possible side effects from treatment. The pros and cons should be discussed before testing.
Q9: What are the stages and grades of prostate cancer?	“Stage” describes how far the cancer has spread: Stage I–II: Cancer is confined to the prostate. Stage III: Cancer has grown through the prostate capsule or into nearby tissues. Stage IV: Cancer has spread to other organs, such as lymph nodes or bones. “Grade” describes how abnormal the cancer cells look under the microscope and how likely they are to grow and spread. The most common grading system is the Gleason score (or Grade Group), usually ranging from: Low grade (Grade Group 1, Gleason 6): slower growing; Intermediate grade (Grade Groups 2–3); High grade (Grade Groups 4–5, Gleason 8–10): more aggressive. Stage and grade together, along with PSA level, help guide treatment choices.
Q10: What are the side effects of prostate cancer treatments?	Side effects depend on the treatment type and the person’s health. Not everyone gets all of these, and many improve over time or can be managed. Common side effects include: Urinary problems: leakage (incontinence), urgency, or weak stream, especially after surgery or radiation; Sexual problems: difficulty getting or keeping erections, changes in ejaculation, and reduced fertility; Bowel issues: looser stools, more frequent bowel motions, or rectal irritation after pelvic radiation; Hormone therapy effects: hot flushes, tiredness, weight gain, loss of muscle, reduced sex drive, mood changes, and thinning of bones; General treatment effects: fatigue, risk of infection and other chemotherapy related effects for those having chemotherapy. Before starting treatment, patients should be told about likely side effects, how long they may last, and what can be done to prevent or treat them.

Supplementary Table S6. Microsoft Copilot Responses to Standardized Prostate Cancer Prompts (Verbatim)

Prompt	Microsoft Copilot Response
Q1: What is the prostate and what does it do?	The prostate is a small gland in the male reproductive system. It sits just below the bladder and surrounds the urethra (the tube that carries urine out of the body). Its main job is to make a fluid that helps form semen, which protects and nourishes sperm.
Q2: What is prostate cancer?	Prostate cancer happens when abnormal cells in the prostate start growing in an uncontrolled way. Sometimes the cancer grows slowly and stays in the prostate; other times it can spread to other parts of the body, such as bones or lymph nodes.
Q3: What are the symptoms of prostate cancer?	Early prostate cancer often causes no symptoms. When symptoms do appear, they may include: Needing to urinate more often, especially at night; Weak or interrupted urine flow; Difficulty starting urination; Pain or burning when urinating; Blood in urine or semen; Pain in the back, hips, or pelvis (more common in advanced cancer). These symptoms can also be caused by non cancerous prostate conditions.
Q4: What is a PSA test and what does PSA mean?	PSA stands for Prostate-Specific Antigen, a protein made by prostate cells. A PSA test is a simple blood test that measures the level of PSA in the bloodstream. Higher-than-expected PSA levels can be a sign of prostate cancer, but they can also be caused by benign prostate enlargement, infection, or even recent ejaculation.
Q5: What are the treatment options for prostate cancer?	Treatment depends on the stage, grade, and overall health of the patient. Common options include: Active surveillance - monitoring low risk cancers closely; Surgery - removal of the prostate (prostatectomy); Radiation therapy - external beam or brachytherapy; Hormone therapy - lowering testosterone to slow cancer growth; Chemotherapy - used mainly for advanced cancer; Targeted therapies or immunotherapy - for specific cancer types. Doctors tailor treatment to each person’s situation.
Q6: How common is prostate cancer in Australia?	Prostate cancer is the most commonly diagnosed cancer in Australian men. In 2025, an estimated 28,868 men will be diagnosed, making up about 30% of all new male cancer cases. Survival is high: the 5 year survival rate is 96%.
Q7: What are the risk factors for prostate cancer, including family history and genetics?	Key risk factors include: Age - risk increases significantly after age 50; Family history - having a father or brother with prostate cancer increases risk; Genetics - inherited gene changes (e.g., BRCA1/BRCA2) can raise risk; Ethnicity - men of African ancestry have higher risk (global data); Lifestyle factors - obesity may increase the chance of aggressive disease.
Q8: Should I be screened for prostate cancer, and when?	Screening usually involves a PSA blood test, sometimes combined with a digital rectal exam (DRE). General guidance (not a personal medical recommendation): Men aged 50-69 are often encouraged to discuss PSA testing with their doctor. Men with a family history may start the conversation earlier (around age 40-45). Screening

Prompt	Microsoft Copilot Response
	decisions should be made with a GP, considering personal risk factors and preferences.
Q9: What are the stages and grades of prostate cancer?	Two key systems describe prostate cancer: Stages (how far the cancer has spread): Stage I-II: Cancer is contained within the prostate; Stage III: Cancer has grown through the prostate capsule; Stage IV: Cancer has spread to lymph nodes, bones, or other organs. Grades (how aggressive the cancer looks under a microscope): The Gleason score or Grade Group describes how abnormal the cells appear. Grade Groups range from 1 (least aggressive) to 5 (most aggressive).
Q10: What are the side effects of prostate cancer treatments?	Side effects depend on the treatment type but may include: Surgery: Urinary incontinence; Erectile dysfunction; Infertility. Radiation therapy: Urinary urgency or discomfort; Bowel changes (loose stools, rectal irritation); Erectile dysfunction. Hormone therapy: Hot flushes; Reduced libido; Weight gain; Fatigue; Bone thinning. Chemotherapy: Fatigue; Hair loss; Increased infection risk. Doctors work with patients to manage and reduce side effects as much as possible.

Supplementary Table S7. Examples of Minimal Actionable Content Observed in Chatbot Responses

Chatbot	Example statement from response	PEMAT-P actionability interpretation
Microsoft Copilot	"Men aged 50–69 are often encouraged to discuss PSA testing with their doctor."	Identifies a potential patient action (discussion of screening) but lacks structured steps, decision aids, or practical instructions.
Perplexity	"Screening should be a shared decision with a doctor."	Suggests consultation but does not provide specific guidance on how to act on the information.
ChatGPT 5.2	"Treatment depends on how risky the cancer is, whether it has spread, and your overall health."	Informational description only with no explicit patient-directed action.
Claude	"Symptoms may include urinary changes or blood in the urine."	Descriptive educational information without guidance on next steps or patient decision-making.
Google Gemini	"PSA testing may be discussed with a doctor depending on risk."	General suggestion rather than actionable instruction with defined steps.
